# Monitoring of severe traumatic brain injury patients in UK ICUs: a national survey

**DOI:** 10.1186/cc12273

**Published:** 2013-03-19

**Authors:** S Panchatsharam, B Lewinsohn, G De La Cerda, D Wijayatilake

**Affiliations:** 1 Queen's Hospital, Romford, UK

## Introduction

Prevention of secondary brain injury is the cornerstone in the management of patients with severe traumatic brain injury (TBI) and raised intracranial pressure (ICP). Although a variety of monitoring methods are available, due to lack of strong evidence their use varies considerably [1]. The objective of this survey was to provide an overview of the current practice in monitoring of patients with severe TBI in all neuro-ICUs across the UK.

## Methods

The ICUs managing adult patients with severe TBI were identified from the RAIN (Risk Adjustment In Neurocritical care) study sites, and the Society of British Neurosurgeons. Thirty-one centers were identified, and a telephonic survey was conducted by the investigators. Data were collected using a single-page questionnaire containing 18 questions.

## Results

All 31 (100%) units used ICP monitoring and parenchymal ICP bolt was the most widely used method (77%). Thirty (96.7%) units used a cerebral perfusion pressure protocol to guide therapy. Twenty-eight (90.3%) units used continuous capnography. Twelve (38.7%) units used transcranial Doppler. Eight (25.8%) units used partial pressure of oxygen in brain tissue. Four (12.9%) units used microdialysis. Only one unit used jugular bulb oximetry. None of the units used near-infrared spectrometry or optic nerve sheath diameter. Eight units (25.8%) used a cerebral function monitor and seven (22.5%) units used the bispectral index for guiding depth of sedation.

## Conclusion

This survey shows that there is no clear consensus on what types of monitoring should be used to guide management of patients with TBI. A CPP protocol, based on measurement of ICP, is the most widely used method, although a recent randomized control trial did not show any benefit in outcome [2]. Other invasive monitoring methods, although they may help in individualized care, are still not yet popular to due lack of strong evidence. Larger multicentre portfolio studies are needed to establish their benefits.

## References

[B1] Wijayatilake Updates in the management of intracranial pressure in traumatic brain injury Curr Opin Anaesthesiol 2012 25 540 547 10.1097/ACO.0b013e328357960a 22914351

[B2] Chesnut A trial of intracranial-pressure monitoring in traumatic brain injury N Engl J Med 2012 367 2471 2481 10.1056/NEJMoa1207363 23234472 PMC3565432

